# Clonal growth characteristics and diversity patterns of different *Clintonia udensis* (Liliaceae) diploid and tetraploid cytotypes in the Hualongshan Mountains

**DOI:** 10.1038/s41598-024-66067-0

**Published:** 2024-07-05

**Authors:** Mian Han, Qiyang Qie, Meilan Liu, Huiqin Meng, Tiantian Wu, Yadi Yang, Lingling Niu, Genlou Sun, Yiling Wang

**Affiliations:** 1 School of Life Science, Shanxi Normal University, Taiyuan, 030031 China; 2 Department of Botany, Saint Mary’s University, Halifax, NS B3H 3C3 Canada

**Keywords:** Evolution, Plant sciences

## Abstract

Polyploidization plays an important role in plant evolution and biodiversity. However, intraspecific polyploidy compared to interspecific polyploidy received less attention. *Clintonia udensis* (Liliaceae) possess diploid (2*n* = 2*x* = 14) and autotetraploid (2*n* = 4*x* = 28) cytotypes. In the Hualongshan Mountains, the autotetraploids grew on the northern slope, while the diploids grew on the southern slopes. The clonal growth characteristics and clonal architecture were measured and analyzed by field observations and morphological methods. The diversity level and differentiation patterns for two different cytotypes were investigated using SSR markers. The results showed that the clonal growth parameters, such as the bud numbers of each rhizome node and the ratio of rhizome branches in the autotetraploids were higher than those in the diploids. Both the diploids and autotetraploids appeared phalanx clonal architectures with short internodes between ramets. However, the ramets or genets of the diploids had a relatively scattered distribution, while those of the autotetraploids were relatively clumping. The diploids and autotetraploids all allocated more biomass to their vegetative growth. The diploids had a higher allocation to reproductive organs than that of autotetraploids, which indicated that the tetraploids invested more resources in clonal reproduction than diploids. The clone diversity and genetic diversity of the autotetraploids were higher than that of the diploids. Significant genetic differentiation between two different cytotypes was observed (*P* < 0.01). During establishment and evolution, *C. udensis* autotetraploids employed more clumping phalanx clonal architecture and exhibited more genetic variation than the diploids.

## Introduction

In plants, polyploidy has long been considered a widespread and natural phenomenon^1–4^. It has been estimated that all angiosperms have polyploid origins^5–7^, approximately 35% of extant flowering plant species have recent polyploid origin^8^. Polyploids usually have different morphological, physiological, or genetic features than their diploid parents^9–13^. These differences frequently resulted in improved adaptation potential and fitness^14–17^. For example, *Ranunculus auricomus* polyploids appeared in a mosaic of intermediate and transgressive phenotype^18^ and had three times higher heterozygosity than their diploid progenitor^19^. A positive association between heterozygosity and temperature seasonality suggested a higher resistance of *R. auricomus* polyploids to more extreme climatic conditions. Adaptive changes, such as increased tolerance to extreme environmental conditions, effective means of vegetative reproduction, apomixis^20^, pest resistance, variation in organ size, flowering time, and biomass, have facilitated the evolutionary success of polyploids^12,16,21–23^. Therefore, polyploids are recognized as important drivers of diversification, distribution and adaptive responses to novel environments^2,6,8,24^.

Polyploids are primarily classified as autopolyploids and allopolyploids^21^. Autopolyploids originate within a single species through whole-genome duplication (WGD) and possess a genome with multiple sets of homologous chromosomes^25^, and play important evolutionary and ecological roles in natural populations^21,23,24,25^. Compared with allopolyploids, autopolyploids display some disadvantages, such as reduced fertility and inbreeding depression. Consequently, autopolyploids have been historically thought as an evolutionary dead end. Although this view has under debate, the biases about the evolutionary potential of autopolyploids have left. Thus, the research on autopolyploids is still far less than that on allopolyploids^24,25–29^.

Currently, there are more studies about autopolyploids, including the influence factors of autopolyploid formation and establishment, the roles in autopolyploid evolutionary success, the differentiation and the ecological niche differentiation between diploids and autopolyploids, even the molecular comparisons of autopolyploidy and allopolyploidy^2,30–35^. However these were mainly focusing on the interspecific autopolyploidy^19,32,35^, the studies on naturally intraspecific autopolyploids still remain rare.

In angiosperms, many perennial flowering plants possess both sexual reproduction and asexual propagation^36–38^. Asexual propagation via clonal growth plays major contributors for the local population growth, high resilience and sink for resources, and through foraging behavior to respond to environmental quality^39^. Sexual reproduction increases the genetic diversity and maintain the genetic differentiation of populations^12,40^. Genetic differentiation can decrease the susceptibility to the competition, diseases and locally adverse environmental conditions^39,41^. Both modes of reproduction have often been viewed as temporally or spatially adaptations to variable environments^2,42^.

*Clintonia udensis*, a perennial herb, belongs to *Clintonia* genus of Liliaceae family, widely distributed across Eastern Asia, including the Himalayas, China, Korea, Japan and the Far East of Russia. This species has two cytotypes that include diploids (2*n* = 2*x* = 14) and tetraploids (2*n* = 4*x* = 28)^36,43–46^. Although the diploids are continuously and extensively distributed from Yunnan Province in South China to Heilongjiang Province in Northern China, the tetraploids are restricted to the Jade Dragon Snow Mountains in Yunnan Province, Shennongjia in Hubei Province, and the Hualongshan Mountains in Shaanxi Province, which overlap, or are adjacent to the distribution areas of diploids.

According to the chromosomal components and karyotypes, the tetraploids of *C. udensis* are regarded as autopolyploids with multiple origin^47^. Within the same population, the chromosome numbers were found to be very stable and there were no different ploidy individuals mixed.

*C. udensis* has a unicellular archesporium acting directly as the megespore mother cell. Its megasporogenesis development is a unique *Clintonia*-type, which is equivalent to female self-fertilization^48,49^. The pattern of the mature embryo-sac development belongs to the tetrosporic highly reduced *Fritillaria* type that contains 5 or 6 nuclei^50^. No apomixes occurred in this species. Both diploids and autotetraploids have a hard rhizome, *C. udensis* not only performs asexual propagation through axillary buds but also generates seeds through sexual reproduction. The pattern of breeding system of this species was facultative xenogamy^51^. In natural habitats, *C. udensis* diploids and autotetraploids can generally produce seedlings and grow in fallen decayed wood and moist mossy ground^51^.

Although there are no significant morphological differences between diploids and autotetraploids, morphological diversity (e.g., leaves, fruits, inflorescences, and pollens) has been observed among different *C. udensis* populations^47,51,52^. Interestingly, only the seed size of autotetraploids is significantly larger than that of diploids^53^. Furthermore, the color of the scape in the diploids is brown, but is green in the autotetraploids^51^. Genetic differentiation occurred among different populations and between different cytotypes^36,43^. Li et al^47^. suggested the autotetraploids of *C. udensis* were currently at a strong differentiated stage and only regarded as an intraspecific autotetraploids.

In the Hualongshan Mountains, *C. udensis* diploids grow primarily at altitudes of 2450 m on the southern slopes, while the autotetraploids grow at altitudes of 1900 m on the northern slopes. In general, polyploids can tolerate harsher environmental conditions than its diploids. Different elevations can lead to heterogeneous habitats. At different altitudes of the same mountains, what would the survival strategy for the diploids and autotetraploids of *C. udensis* adopt? How is clonal architecture and what are diversity levels of different cytotypes?

To solve the above problems, we analyzed the clonal configurations and clonal diversity of the autotetraploids and diploids to reveal the genetic diversity and differentiation between two different ploidy cytotypes and elucidate the possible reasons for the variation in *C. udensis* different cytotypes. Our results provide valuable knowledge for understanding natural intraspecific autopolyploidy.

## Materials and methods

### Sample sites and materials

The samples were collected from the Hualongshan National Nature Reserve in Shaanxi Province, China, with geographic coordinates of 109°16′41″–109°30′29″ E, 31°54′39″–32°08′13″ N. The highest peak of the Hualongshan Mountains is 2917 m above sea level, which is the second highest peak of the Dabashan mountain. Based on the vegetation regionalization of China, the Hualongshan Mountains belong to subtropical evergreen broadleaved forest, north subtropical evergreen forest, and deciduous broadleaved mixed forest areas^52^.

In the Hualongshan Mountains, the diploids grew on the shady southern slopes, whereas the autotetraploids grew under the arbor forests on the northern slopes^51^. The annual average temperature and mean annual precipitation is about 6.9 °C and 1171.5 mm in the southern slopes, while approximately 10.7 °C and 813.5 mm in the northern slopes, respectively^36,43^.

Our field survey was conducted in accordance with the laws of the People’s Republic of China, and sample collection was approved by the Chinese Government. All researchers received the permission letters from the College of Life Science, Shanxi Normal University. The voucher specimens were deposited in the herbarium of College of Life Science, Shanxi Normal University (NO: 201705001-201705050).

**Figure 1:**
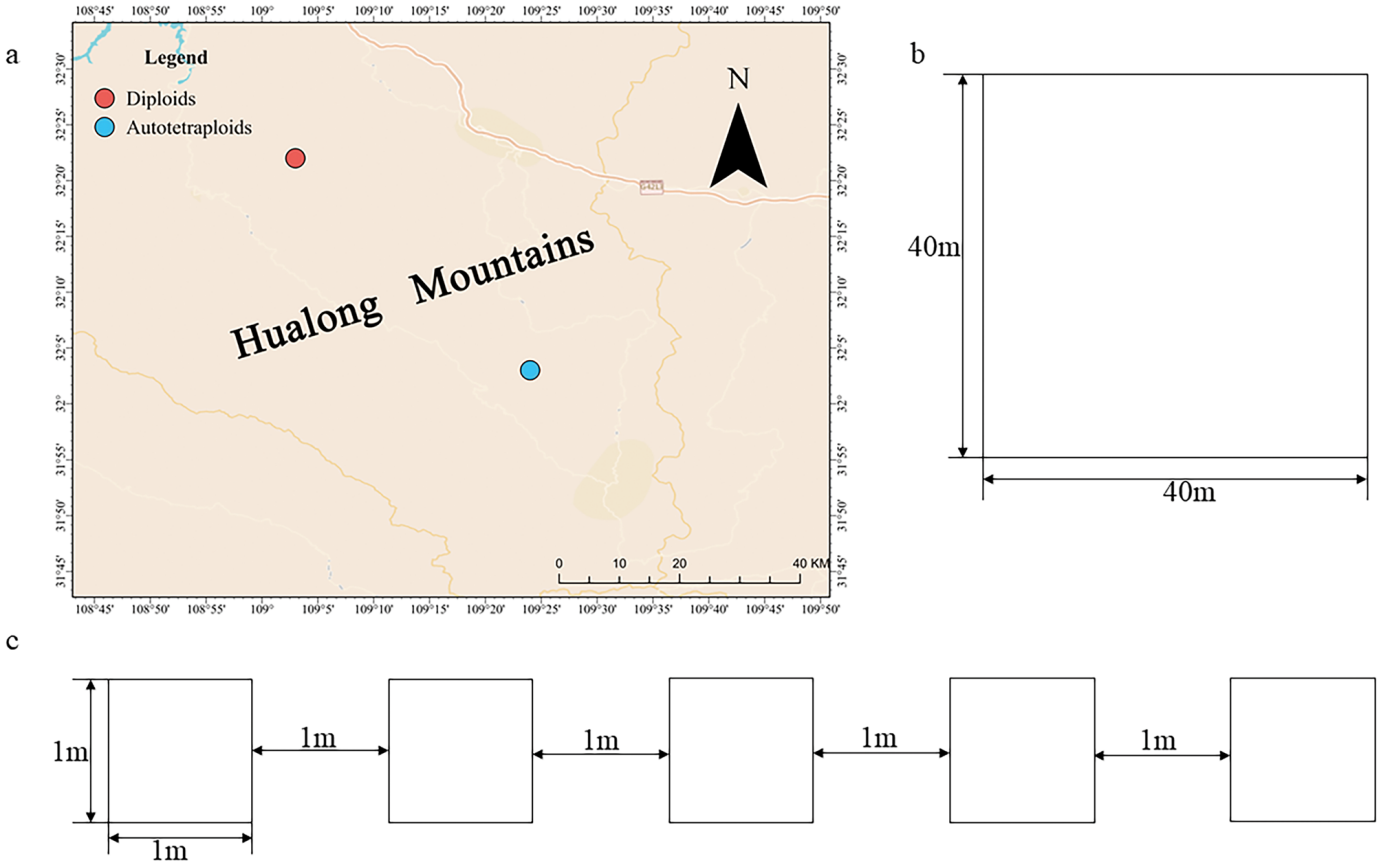
The quadrat designed of *Clintonia udensis* in Hualong Mountains (**a** the sampled sites; **b** the sampled plot for the diploids and autotetraploids respectively; **c** 1 m × 1 m squares by the contiguous grid quadrat designed).

**Figure 2:**
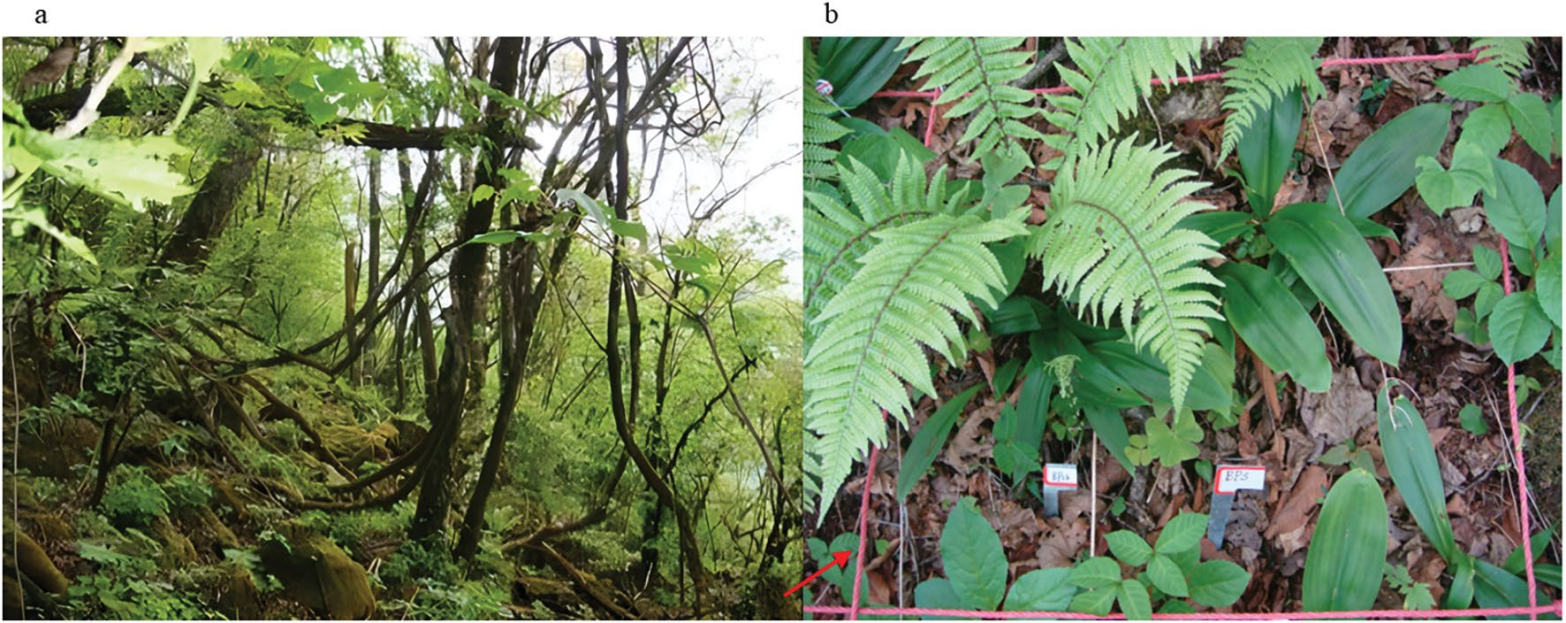
The habitats and designed squares (**a** the habitats of *Clintonia udensis*; **b** one of the designed squares, the red box was a 1 m × 1 m square).

Using the quadrat methods, two > 800 m^2^ plots (Fig. 1a,b) were established respectively in the southern and northern slopes of Hualongshan Mountains. Each plot was subsequently divided into 625 1 m × 1 m squares by the contiguous grid quadrat with cords^54^ (Fig. 1c). Then, ten 1 m × 1 m squares were randomly chosen for the diploid and tetraploid, respectively (Fig. 2). Within the sampled ten squares, a total of 93 mature diploid and 107 tetraploid individuals were counted. And the specific spatial positions of each sampled individual of the two ploidies were marked and recorded (Table S1).

### Ploidy identification of sampled individuals

Root tips were collected from each sampled individual and fixed in FAA (Formalin-Aceto-Alcohol) for observing chromosomal numbers. The specimens were firstly prepared according to the methods of Yang (1997). Subsequently, chromosome numbers were counted with an optical microscope to confirm the ploidy level of the studied individuals.

### Clonal growth and configuration analysis

**Figure 3:**
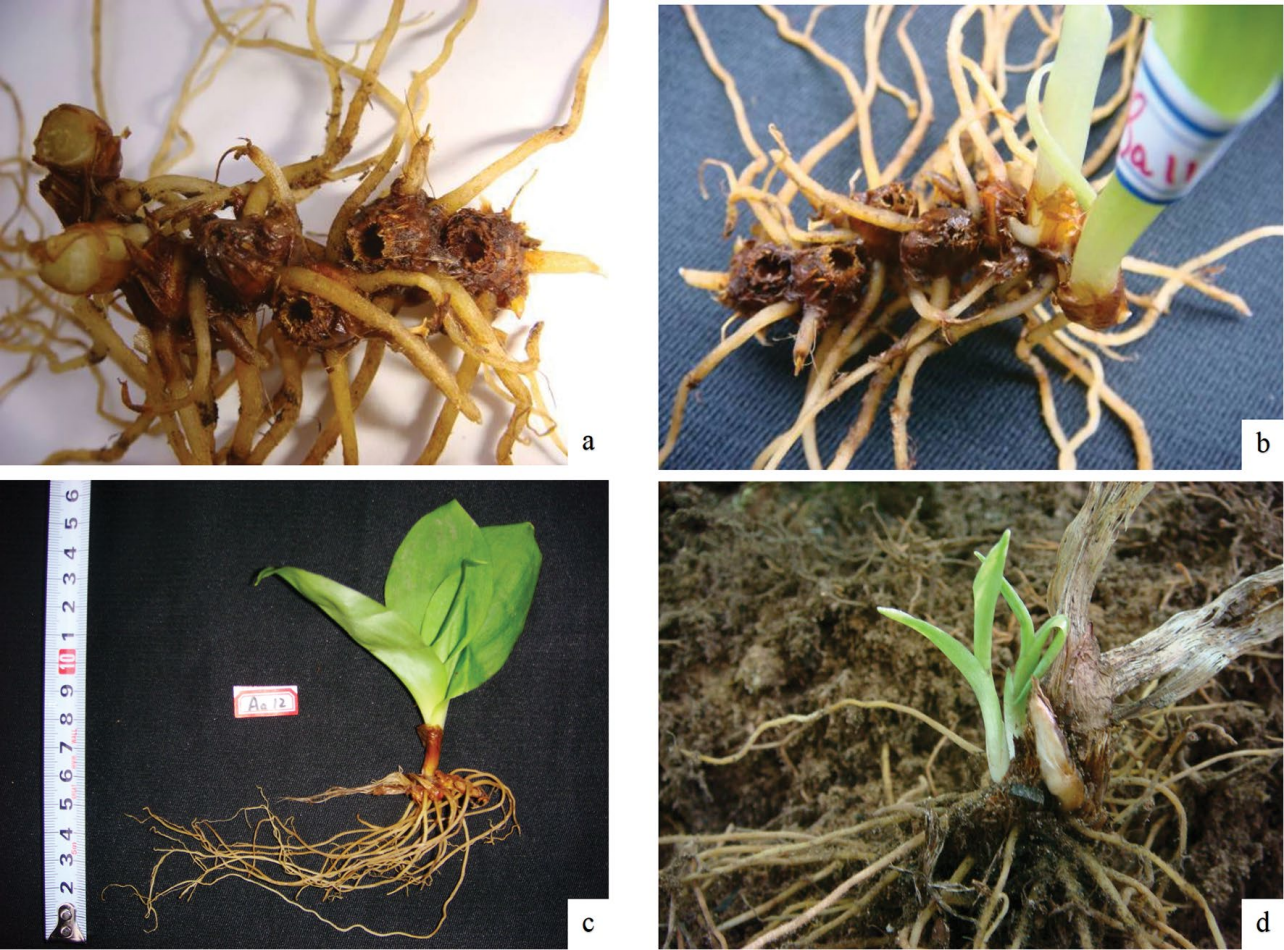
Rhizomes and rosette seedings of *Clintonia udensis* (**a** rhizomes; **b** rosette seedings; **c** the individual of from rhizomes; **d** the seeding from rhizomes).

Each sample individual was transferred to the laboratory. Then the soil was removed from the roots of samples. When the roots were completely uncovered, the rhizome nodes (Fig. 3) were measured. In each growing season per year, the numbers of *C. udensis* rhizome nodes are added. Thus, the number of buds (active and dormant buds) on each rhizome, the ages and numbers of branches of the rhizomes were quantified. The primary buds on the rhizomes were marked as one, which represented the branch development in the first year. Subsequently, the branches developed from the following year were marked as two, and so on (e.g., the branches of the third year were marked as three).

Meanwhile, the biomass of different modules, including vegetative organs biomass, reproductive organs biomass, underground biomass and total biomass, were weighed after these parts and were oven-dried at 70 °C for 48 h. The morphological characteristics, for example flowers, fruits and seeds, were observed and recorded (Fig. S1).

The mean value of clonal growth parameters was calculated with Excel software. The obtained average data from all sampled individuals of diploids and autotetraploids was analyzed through one-parametric ANOVA (analysis of variance) using SPSS v 20 software. LSD (least-significant difference) was employed to examine the significance among different groups.

Additionally, the coefficient of variation (*CV*) was calculated with the formula *CV* = the standard deviation/the average value.

### Simple sequence repeat (SSR) analysis

The leaves of all the sampled individuals were removed with scissors, dried using silica gel, and stored at – 80 °C. The total genomic DNA was then extracted from the leaf samples following a modified CTAB method^43^. The quality of the extracted DNA was determined using electrophoresis in a 1% agarose gel, and the concentration was quantified using an UV nucleic acid analyzer.

According to the SSR molecular markers from the EST (expressed sequence tag) data of Liliaceae^45^, eight pairs of primers with clear amplification bands and good repeatability were screened out from 18 SSR pairs of primers (Table S2). Subsequently, all of the collected *C. udensis* individuals were amplified by PCR using the selected primers.

The PCR reaction volume was 10 μL, including 5 μL Mix, 1 μL of DNA template, 0.6 μL of each SSR primer, and 3.4 μL of ddH_2_O. The reaction procedure was as follows: pre-denaturation at 94 °C for 5 min, 35 cycles of denaturation at 94 °C for 30 s, annealing at 45 °C–60 °C for 30 s, extension at 72 °C for 2 min, final extension at 72 °C for 5 min^45^. The PCR products were finally stored at − 4 °C. The amplified products were separated using 10% denaturing polyacrylamide gel electrophoresis and visualized using silver staining^55^.

### Clonal diversity analysis

The clonal diversity in both diploids and autotetraploids was assessed through several parameters, including total genet number (*G*), average clone size (*N*/*G*), Simpson index (*D*), and Fager index (*E*)^55,56^. The calculation formulas for the Simpson and Fager indices were referenced from Wang et al^43^.

### Genetic diversity and differentiation analysis

According to the previous studies^12,57,58^, a band observed in the samples received a score of 1 at a particular site, while its absence at the same site was marked as 0. Finally, a binary matrix comprising 51 loci was generated and documented.

Using GENEPOP v3.1 software^59,60^, the linkage disequilibrium of each locus was firstly detected and the loci that presented linkage disequilibrium were removed (*P* < 0.001). Then, Nei's gene diversity index (H), Shannon phenotype information index (I), and the percentage of polymorphic bands (*PPL*) were calculated using the POPGENE v 1.3.1 software package^61^.

The differentiation coefficient (*G*_ST_) was also estimated by POPGENE to explore the differentiation between diploids and autotetraploids. Thedegree of molecular variation and the distribution pattern of genetic variation between different cytotype were further analyzed using ARLEQUIN v 3.11 software^62,63^.

## Results

### Ploidy level of the sampled individuals

Based on the observation, the chromosome number of sampled individuals was fourteen in the southern slope, twenty-eight in the northern slopes, which is diploid and tetraploid, respectively (Fig. S2).

### Clonal characteristics and spatial architectures of diploids and tetraploids

The average number of buds on each diploid rhizome was 2.120, and the variance degree of all buds was 0.277 in the diploids (Table 1). For the autotetraploids, the average number and variance of buds per rhizome were 2.315 and 0.408, respectively. The proportion of active buds to total buds was 0.627 for the diploids and 0.473 for the autotetraploids. The ratio of dormant buds to total buds for the diploids was 0.357, while it was 0.522 for the autotetraploids.

**Table 1:** Rhizome buds of *Clintonia udensis* diploids and autotetraploids.

		Number	Mean value and standard deviation	Variation of rhizomes among sampled individuals
Bud number of each rhizome of all sampled individuals	2x	1–4	2.120 ± 0.5260	0.277
	4x	1–4	2.315 ± 0.6391	0.408
Mean number of rhizome buds of all sampled individuals	2x	39	2.133 ± 0.4714	0.222
	4x	39	2.283 ± 0.4031	0.163
Active buds of rhizomes of all sampled individuals	2x	39	0.627 ± 0.1899	0.036
	4x	39	0.473 ± 0.7984	0.006
Dormant buds of rhizomes of all sampled individuals	2x	39	0.357 ± 0.2047	0.042
	4x	39	0.522 ± 0.7422	0.006

One-way ANOVA analysis showed that the above indices, such as the active buds and the dormant buds, were significantly different between the *C. udensis* diploids and autotetraploids (*P* < 0.05) (Table S3). The autotetraploids possessed more rhizome buds and higher dormant buds than those in diploids, however, the number of active buds were lower than that in the diploids (Table 1).

**Figure 4:**
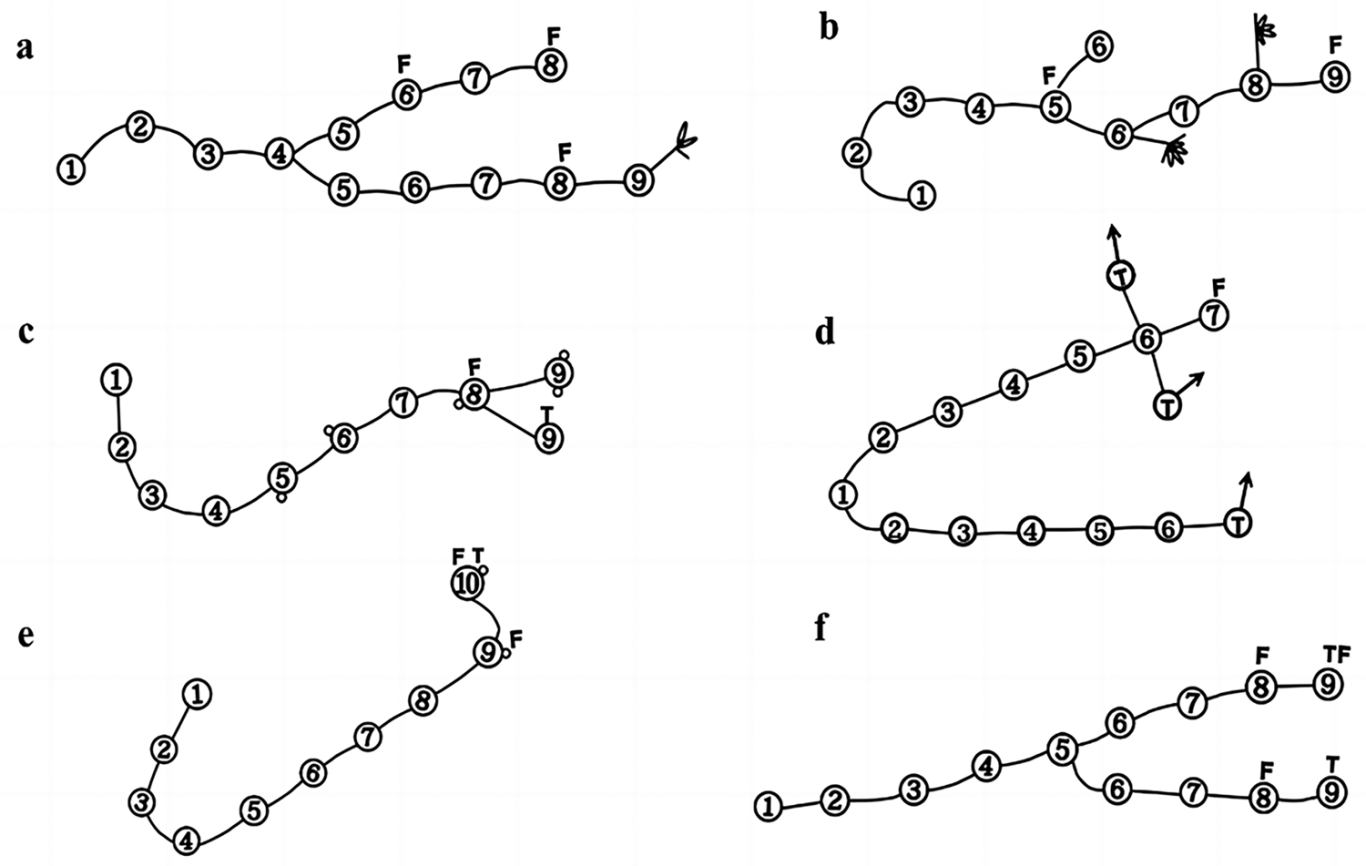
Clonal architecture of *Clintonia udensis* (**a** the clonal architecture of diploids; **b** the clonal architecture of autotetraploids; **c**,**d** different type of clonal architecture: **c** zigzag type; **d** C type; **e** V type; **f** Y type). The number in the figures represented for years. Such as ①first year; ②second year…… etc. F, represented the flowering node; T, represented the terminal, namely the maximum age of the samples.

The rhizome branch types of the diploids and autotetraploids were found to be primarily zigzagged, C, V, and Y (Fig. 4), where the "one" type was very rare. The spatial positions among the buds of each rhizome basically presented a symmetrical distribution when there were two buds, or formed 90° angles when having 3–4 buds.

For the *C. udensis* diploids and autotetraploids, the lengths of rhizome internodes (namely spacer) were from ~ 1 to 2 cm (Fig. 3). The rhizome buds typically occurred at the rhizome node, which regarded as a ramet produced by the genet. The number of branches was generally from one to four, among which one branch with the highest frequency, and the other branch number were usually observed in older individuals.

**Figure 5:**
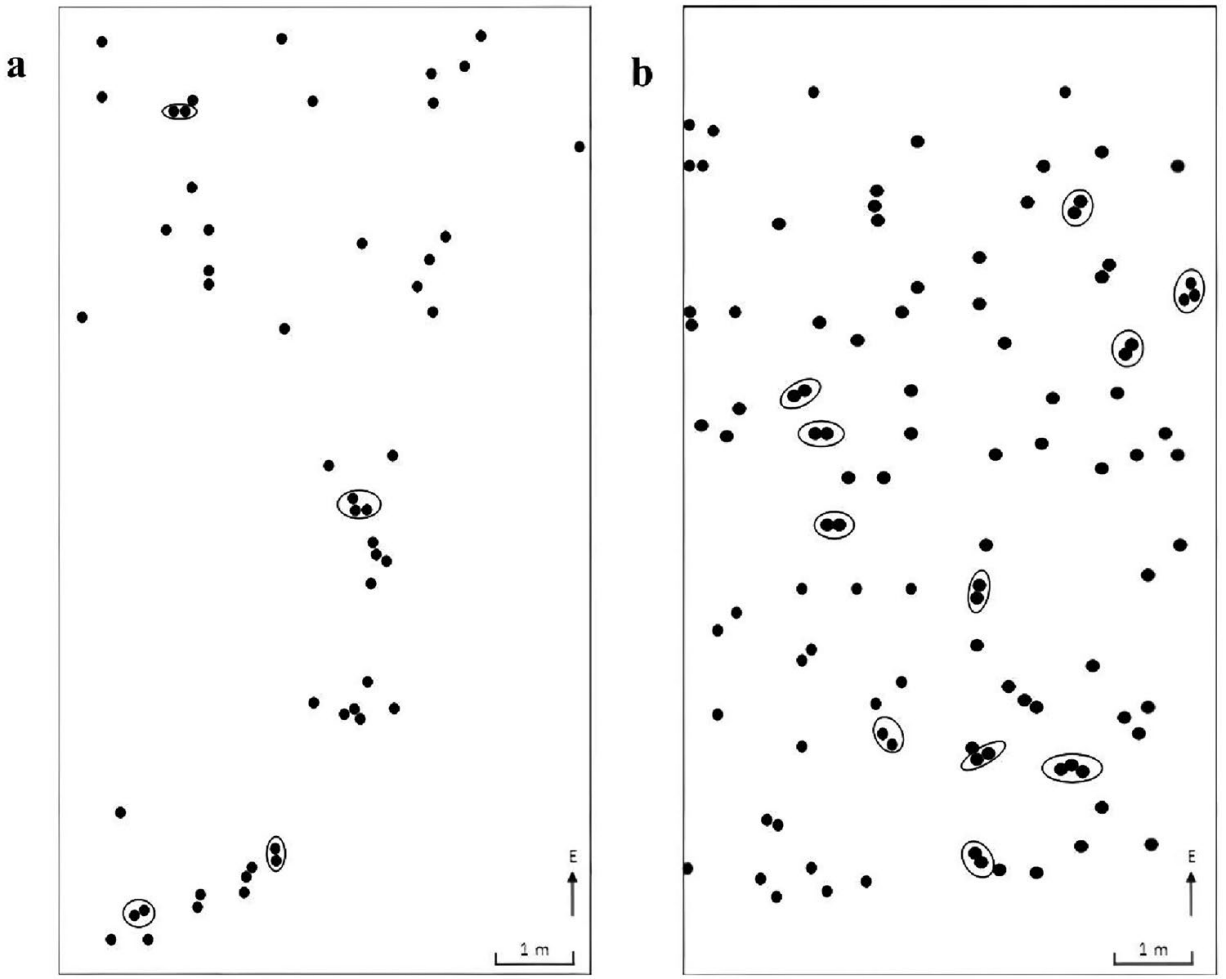
Phalanx clonal architecture of *Clintonia udensis* (a, diploids; b, autotetraploids). Black dots presented sampled individual; In the black circle, each dot presented a ramet. For the diploids, the ramet individuals were relatively less and far away each other than that of the autotetraploids.

The number of ramets produced by both diploid and tetraploid individuals of *C. udensis* was very limited. The ratio of clone individuals was relatively low for both cytotypes. Thus, the clone growth pattern of *C. udensis* diploids and autotetraploids was suggested to be the phalanx form (Fig. 5).

For biomass perspective, the biomass allocated to vegetative growth was higher than to reproduction both in the diploids and tetrapliods. The underground biomass of diploids was 0.554, which lower than that of tetraploids with a mean 1.516. And the total biomass of diploids also was lower than that of tetraploids. Totally, the different modules biomass of tetraploids were all higher than that of diploids (Table 2). At the reproduction allocation, the proportion of diploids was 22.26 while that of tetraploids was 16.08, showed the diploids had a higher allocation to reproductive organs than that of the tetraploids (*P* < 0.001).

**Table 2:** The biomass of diploidy and autotetraploidy of *Clintonia udensis.*

Biomass		Min	Max	Average	Standard deviation	*CV*	*P*
Total biomass (g)	2*x*	0.0085	5.203	1.474	1.008	0.683	0.0813
	4*x*	0.057	7.55	3.116	2.507	0.804	
Nutrient organs (g)	2*x*	0.727	2.473	1.383	0.508	0.367	0.1157
	4*x*	1.700	7.282	3.307	1.632	0.418	
Reproductive organs (g)	2*x*	0.147	1.104	0.369	0.244	0.662	0.0252**
	4*x*	0.327	1.878	0.825	0.489	0.592	
Underground parts (g)	2*x*	0.007	1.379	0.554	0.311	0.561	0.1185
	4*x*	0.028	4.109	1.516	1.201	0.791	
Reproductive allocation	2*x*	5.890	48.72	22.26	0.104	0.467	0.0007***
	4*x*	1.080	37.45	16.08	0.098	0.591	

Based on *CV* values, only the reproductive organs biomass of the tetraploids was lower than that of diploids, which indicated this trait was relatively stable than that of the diploids.

### Clonal diversity and structures of diploids and tetraploids

No dominant clones were found in the *C. udensis* diploids and autotetraploids. The diploids consisted of 46 genotypes (genets), of which three genets had two clonal ramets, and one genet had three clonal ramets, while the other 42 genets had only a single clonal ramet. For the autotetraploids, there were 89 genotypes (genets), of which three genets had two clonal ramets, and two genets had three clonal ramets, while the other 78 genets had only a single clonal ramet (Fig. 5). Further, there were no identical genotypes were found among the differentploidy individuals, which indicated that the differentiation occurred between the diploids and autotetraploids.

Compared with the autotetraploids, the numbers of clones (genets) of the diploids were relatively lower. The spatial distribution of clones or genets was scattered in the diploids. whereas was relatively clumping in the autotetraploids (Fig. 5). The clonal propagation characteristics were different between the diploids and autotetraploids.

Meanwhile, no shared genotypes were found between the autotetraploids and diploids (Table 3). The average clone size (*N*/*G*) of the tetraploids was 1.146, whereas the Simpson diversity (*D*) and Fager evenness (*E*) indices were 0.4695 and 0.6863, respectively. Correspondingly, these parameters for the diploids were 1.109, 0.3741, and 0.4612 respectively. There were significant differences in clonal diversity between the diploids and tetraploids (*P* < 0.05) based on Simpson diversity (*D*) and Fager evenness (*E*) indices. These results showed that the autotetraploids had higher clonal diversity than the diploids.

**Table 3:** Genetic diversity and differentiation of the diploids and autotetraploids of* Clintonia udensis.*

	Total number of loci	Number of polymorphic loci	*PPL*%	H	I	*N*	*G*	*N/G*	*D*	*E*	*G*_ST_
2*x*	51	32	63.5	0.2127	0.3012	51	46	1.1087	0.3741	0.4612	0.6139 (*P* = 0.0023)
4*x*	51	37	68.5	0.2332	0.3416	102	89	1.1460	0.4695	0.6863	
Total	51	40	78.4	0.2358	0.3552	76.5	67.5	1.1274	0.4218	0.5737	

*PPL* the percentage of polymorphic loci, *H* Nei’s gene diversity, *I* Shannon’s information index, *N* sample size, *G* number of genotypes, *N*/*G* average size of genotype, *D* Simpson index, *E* Fager index, *G*_ST_ genetic differentiation coefficient among diploids and autotetraploids.

### Genetic diversity and differentiation of diploids and autotetraploids

All sampled individuals of the *C. udensis* diploids and autotetraploids were genotypedusing SSR markers (Table S2). No significant linkage disequilibrium in the diploids and autotetraploids individuals was found.

Among the 51 clear and stable bands amplified from eight pairs of SSR primers, 40 were polymorphic. At the species level, the percentage of polymorphic loci (*PPL*) was 78.4%, the Nei's gene diversity index H was 0.2358, and the Shannon information diversity index I was 0.6337, which showed a high level of genetic diversity in *C. udensis* (Table 3).

At the ploidy level, the three genetic diversity indices of the autotetraploids were *PPL* = 68.5%, H = 0.2332, and I = 0.3416, while for the diploids they were *PPL* = 63.5%, H = 0.2127, and I = 0.3012 (Table 3). The results revealed that the genetic diversity of the autotetraploids was significantly higher than that of the diploids (*P* < 0.05).

The gene differentiation coefficient *G*_ST_ between the diploids and autotetraploids was 0.6193, which indicated that 61.93% of the total genetic diversity existed between ploidies, and the genetic variation within different ploidies was 38.07% (Table 3). This was consistent with the results of AMOVA analysis, namely, the highest genetic variation occurred between the diploids and the autotetraploids (Table 4).

**Table 4:** Genetic variations of *Clintonia udensis* diploids and autotetraploids.

Source	df	SS	MS	Est. Var	Variance percentage %	Fixation indices
Among groups	1	501.832	51.698	1.959	60.87	*Φ*_CT_ = 0.6089 (*P* = 0.001)
Among populations within groups	17	378.867	39.803	0.923	28.66	*Φ*_ST_ = 0.5732 (*P* = 0.001)
Within populations	311	78.371	17.472	0.337	10.47	*Φ*_SC_ = 0.3654 (*P* = 0.001)

*Φ*_CT_: genetic differentiation between groups; *Φ*_SC_: genetic differentiation between populations within groups; *Φ*_ST_: genetic differentiation between populations.

## Discussion

### Clonal growth differences and architecture of the *C. udensis* diploids and autotetraploids in the Hualongshan Mountains

For *C. udensis*, only a single active bud was found on many rhizomes in both the diploids or the autotetraploids. During the normal growth and development, *C. udensis* individuals develop aboveground components mainly through one active bud on each rhizome node each year. Besides, there were 2–4 active buds on some rhizome nodes. These active buds could grow throughout the year and formed the basal rosette seedlings (Figs. 2, 3).

Newly sprouted rhizomes first formed "one" type of new branches. Each new branch continued to sprout new buds during the subsequent growth season (Fig. 3). These new branches subsequently extended along different direction from the original branches of the former rhizome. Thus, the active buds on each rhizome node formed a symmetrical or 90° angles. Furthermore, the newly formed branches continued to grow and produce additional rhizome nodes. Under this situation, different buds can occupy different spatial positions and develop multiple ramets^64^.

Before the winter, the buds on the rhizome nodes had been developed and formed. When in the autumn and winter, the aboveground parts of *C. udensis* individuals would die, the rhizomes and buds continue to live underground. During the spring, the active buds begin to germinate and form caespitose seedling sprouts, while the dormant buds continuously keep the dormant state^65^.

Between the rhizome internodes, the distances (namely spacers) of *C. udensis* were relatively short (1–2 cm). Short spacer distances made the ramets grow adjacent to each other, which led to a more intensive clone patchiness in the habitats and thus the spatial distribution appeared a phalanx pattern (Figs. 3, 5). In a spatially heterogeneous habitat, shortening spacers may potentially position more newly produced ramets to efficiently acquire resources and thereby to increase survival^20^.

Under different conditions, the bud number and ramet form both changed^20^. For example, *Leymus secalinus* produces spreading ramets (guerrilla form) under low nutrient supply and clumping ramets (phalanx form) under high nutrient supply^20^. Due to the continuous expansion of rhizomes, *C. udensis* gradually formed from a phalanx to a relatively scattered pattern^69^. This change should meet the demands for the space and nutrition of *C. udensis*. With ISSR markers, it has been suggested that the clonal architecture of *C. udensis* may be a guerilla type^36,43^. These showed a trade-off might occur between the phalanx and guerilla forms. To maintain the population development and stabilization, the diploids and autotetraploids occupy and utilize environmental resources through altering ramet form^36,43–45,66^.

According the biomass allocation, the diploids and autotetraploids of *C. udensis* allocated a greater proportion to nutrient growth, which could facilitate their clonal reproduction and growth to enhance the individual competitiveness^67,68^. At the same time, the difference occurred in reproductive allocation between the two cytotypes (Table 2, *P* < 0.01), which indicated the diploids and autotetraploids both had a tendency towards clonal reproduction, and the autotetraploids had a higher clonal growth than the diploids (Table 2).

Within a limited resource pool, if more biomass to the reproductive organs, the allocation to nutrients organs (such as leaves and roots), reduced, which would lead to the decreased ability to obtain resources and affect the individuals survival and growth. When the resources were insufficient, plants could allocate more resources to the nutruent organs to enhance resource acquisition capability. Compared with the ancestral diploids, the autotetraploids allocated more resources to nutrient parts (roots, stems, and leaves) in the relatively poor low altitude environment.

Thus, when the autotetraploids of *C. udensis* formed^47^, they explore the surroundings that different from the diploids. The autotetraploids might respond to the different environment through a higher level of clonal growth than its ancestral diploids, and grow at relatively lower altitudes than that of diploids in the Hualongshan Mountains.

### Diversity and genetic differentiation of *C. udensi*s diploids and autotetraploids in the Hualongshan Mountains

The genetic diversity of *C. udensis* species and different ploidy cytotypes was all high in the Hualongshan Mountains (Table 3). This not only confirmed the biological characteristics of the species, but was consistent with previous studies^36,43–46^.

Under natural conditions, the rate of seed setting of *C. udensis* was 58%, however, the rate of seed setting was 82% through artificial xenogamy^51^. The self-pollination was fertile although the seed setting rate was very low^51^. Therefore, the breeding system of *C. udensis* was facultative xenogamy and pollinated insects were necessary. For the facultative plants, sexual reproduction can supply the loss of genetic variation and increase the level of genetic diversity within the population^11,22,69^.

The clonal propagation of species affected the genetic diversity and population structures^70,71^. And clonal propagation increased the survival of offspring, saved on the resource consumption required for sexual reproduction and impacted the fitness and the evolution of the population^46^. Different clones had the capacity to occupy different microhabitats within heterogeneious habitats. Both the diploids and autotetraploids of *C. udensis* had relatively high clonal diversity (Table 2). *C. udensis*’ rhizomes could place their clonal ramets into favorable environments and renew the buds. These ramets and buds provided resources for seedling formation, ramet growth, and the long-term survival of dormant buds of *C. udensis*’s diploids and autotetraploids^36,43^.

At the same time, a high degree of clonal diversity was maintained due to the quite long lifecycles of clonal plants even under very low levels of seedling regeneration^36,43^. As a perennial herb, this biological characteristic may be a reason for the high clonal diversity of *C. udenesis*. Nevertheless, the clonal diversity of different *C. udensis* cytotypes in the Hualongshan Mountains (Table 3) was lower than the average values of other clonal plants (D = 0.62)^72^, which may be related with the facultative reproduction system of the diploids and autotetraploids.

On the other hand, the level of genetic diversity of autotetraploids of *C. udensis* was higher than that of diploids (Table 3). This was consistent with the previous studies of other species^57,58^. The genetic effective size within a population of clonal plants mainly depends on the genet number, not on the ramet number^73^. The autotetraploids had more genets than the diploids (Table 3), which would explain the higher level of genetic diversity in the autotetraploids of *C. udensis*.

Habitat differentiation is typically regarded as a driver of genetic differences^74^. The autotetraploids occupied a different surrounding compared with its ancestor diploids, a low altitude habitat on the northern slopes in the Hualong Mountains. The different habitats made the autotetraploids of *C. udensis* possessed different clonal and genetic diversity than the diploids. These results were supported the views proposed by Wang^36,43^, but different from that of He^46^.

Furthermore, significant genetic differentiation occurred between the diploids and autotetraploids of *C. udensis* (Tables 3, 4). Based on our field observations, the flowering time of the diploids was about two weeks later than that of the autotetraploids. The fruits of the diploids matured in early August, while the fruits of the autotetraploids matured in early September^51^. The growth cycle of the diploids was ~ 45 days shorter than that of the autotetraploids. These nonsynchronization effectively blocked gene exchange between the diploids and autotetraploids, which enhanced the differentiation between the two cytotypes. Besides, most of the *C. udensis* diploid and tetraploid genotypes were localized; thus, obvious clonal differentiation was observed between the two ploidy cytotypes, which was consistent with the results of Wang et al^36,43^ and He et al^46^. Under different selection pressures, different genotypes would be fixed within the diploids and autotetraploids, respectively, through the formation of different clones^75^.

## Conclusion

In Hualong Mountains, the clonal architectures of different *C. udensis* cytotypes were both represent a phalanx form (clumping ramets). However, the pattern gradually changed from phalanx to scattered during the development. Two cytotypes both allocated more biomass to vegetative growth. The tetraploids inclined towards clonal reproduction, and invested more resources than the diploids. Meanwhile, the autotetraploids exhibited more clonal propagation and higher genetic diversity than their diploid ancestors^76,77^, which potentially make it better use of spatial heterogeneity in resource supply, even at small scales.

### Supplementary Information

Supplementary Figure S1.

Supplementary Figure S2.

Supplementary Tables.

## Data Availability

The data that supporting the findings of this study are openly available in Science Data Bank at https://www.scidb.cn/s/NRJRJn (10.57760/sciencedb.02298).
