# Supplementary material for: Clonal growth characteristics and diversity patterns of different Clintonia udensis (Liliaceae) diploid and tetraploid cytotypes in the Hualongshan Mountains
Source: Sci Rep. 2024 Jul 5;14:15509. doi: 10.1038/s41598-024-66067-0 (PMC11226640; doi:10.1038/s41598-024-66067-0)
Supplement: Supplementary file 1 — Supplementary Figure S1. [file 41598_2024_66067_MOESM1_ESM.pdf]

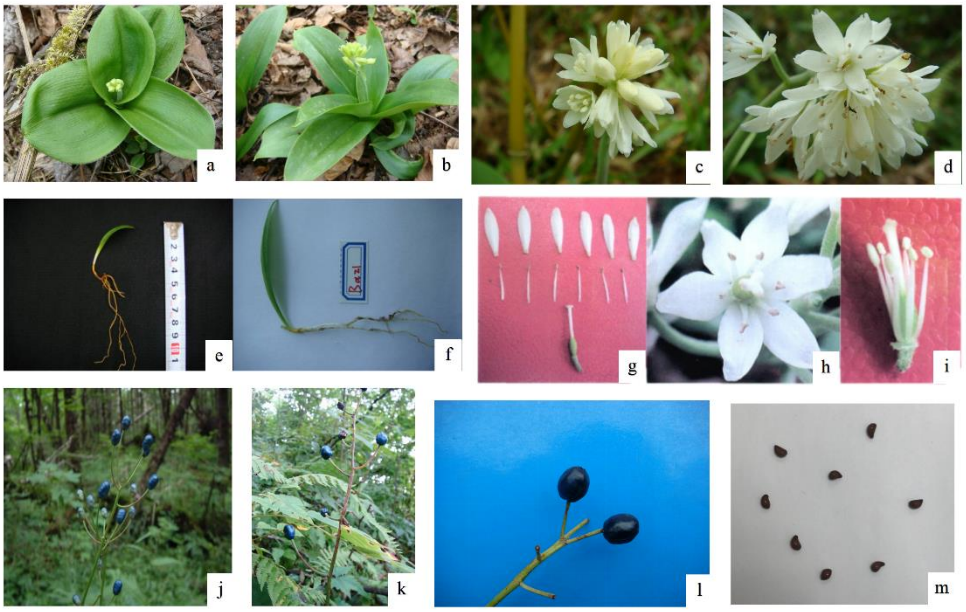

Figure S1. The general morphological traits of *Clintonia udensis* (a, the individual of diploids; b, the individual of autotetraploids; c, flowers; d, the blooming flowers; e, the seeding of diploids; f, the seeding of autotetraploids; g, flower anatomy; h, one flower; i, flower without petals; j, the fruits of diploids; k, the fruits of autotetraploids; l, the mature fruits; m, the seeds).
