# Supplementary material for: Clonal growth characteristics and diversity patterns of different Clintonia udensis (Liliaceae) diploid and tetraploid cytotypes in the Hualongshan Mountains
Source: Sci Rep. 2024 Jul 5;14:15509. doi: 10.1038/s41598-024-66067-0 (PMC11226640; doi:10.1038/s41598-024-66067-0)
Supplement: Supplementary file 2 — Supplementary Figure S2. [file 41598_2024_66067_MOESM2_ESM.pdf]

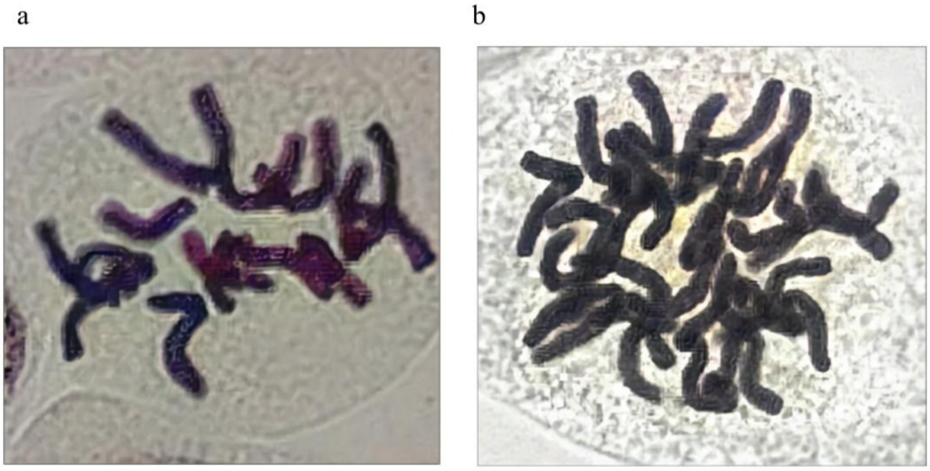

Figure S2. The caryotype of *Clintonia undesis* (a, the chromosome number of sampled individuals in the southern slope (2*x* = 14); b, the chromosome number of sampled individuals in the northern slope (4*x* = 28)).
