# Supplementary material for: Clonal growth characteristics and diversity patterns of different Clintonia udensis (Liliaceae) diploid and tetraploid cytotypes in the Hualongshan Mountains
Source: Sci Rep. 2024 Jul 5;14:15509. doi: 10.1038/s41598-024-66067-0 (PMC11226640; doi:10.1038/s41598-024-66067-0)
Supplement: Supplementary file 3 — Supplementary Tables. [file 41598_2024_66067_MOESM3_ESM.docx]

| Table S1 The information of the sampled individuals | | | | | | | | |
| --- | --- | --- | --- | --- | --- | --- | --- | --- |
| No. | Age | Flowering or no for the year | Ploidy level | Sample site | Voucher ID | Longitude | Latitude | Altitude(m) |
|  |  |  |  | Southern slope |  | 109°03′ | 31°82′ | 2100 |
| a1 | 5 | F | 2x | Southern slope | 201705001 |  |  |  |
| a2 | 7 | F | 2x | Southern slope | 201705002 |  |  |  |
| a3 | 6 | F | 2x | Southern slope | 201705003 |  |  |  |
| a4 | 5 | F | 2x | Southern slope | 201705004 |  |  |  |
| a5 | 5 | F | 2x | Southern slope | 201705005 |  |  |  |
| a6 | 5 | F | 2x | Southern slope | 201705006 |  |  |  |
| a7 | 6 | F | 2x | Southern slope | 201705007 |  |  |  |
| a8 | 11 | F | 2x | Southern slope | 201705008 |  |  |  |
| a9 | 8 | F | 2x | Southern slope | 201705009 |  |  |  |
| a10 | 9 | F | 2x | Southern slope | 201705010 |  |  |  |
| a11 | 7 | F | 2x | Southern slope | 201705011 |  |  |  |
| a12 | 5 | F | 2x | Southern slope | 201705012 |  |  |  |
| a13 | 8 | F | 2x | Southern slope | 201705013 |  |  |  |
| a14 | 5 | F | 2x | Southern slope | 201705014 |  |  |  |
| a15 | 13 | F | 2x | Southern slope | 201705015 |  |  |  |
| a16 | 7 | F | 2x | Southern slope | 201705016 |  |  |  |
| a17 | 8 | F | 2x | Southern slope | - |  |  |  |
| a18 | 8 | F | 2x | Southern slope | - |  |  |  |
| a19 | 5 | F | 2x | Southern slope | - |  |  |  |
| a20 | 7 | F | 2x | Southern slope | - |  |  |  |
| a21 | 6 | F | 2x | Southern slope | - |  |  |  |
| a22 | 5 | F | 2x | Southern slope | - |  |  |  |
| a23 | 5 | F | 2x | Southern slope | - |  |  |  |
| a24 | 5 | F | 2x | Southern slope | - |  |  |  |
| a25 | 6 | F | 2x | Southern slope | - |  |  |  |
| a26 | 11 | F | 2x | Southern slope | - |  |  |  |
| a27 | 8 | F | 2x | Southern slope | - |  |  |  |
| a28 | 9 | F | 2x | Southern slope | - |  |  |  |
| a29 | 7 | F | 2x | Southern slope | - |  |  |  |
| a30 | 5 | F | 2x | Southern slope | - |  |  |  |
| a31 | 8 | F | 2x | Southern slope | - |  |  |  |
| a32 | 5 | F | 2x | Southern slope | - |  |  |  |
| a33 | 13 | F | 2x | Southern slope | - |  |  |  |
| a34 | 7 | F | 2x | Southern slope | - |  |  |  |
| a35 | 8 | F | 2x | Southern slope | - |  |  |  |
| a36 | 8 | F | 2x | Southern slope | - |  |  |  |
| a37 | 7 | F | 2x | Southern slope | - |  |  |  |
| a38 | 5 | F | 2x | Southern slope | - |  |  |  |
| a39 | 7 | F | 2x | Southern slope | - |  |  |  |
| a40 | 6 | F | 2x | Southern slope | - |  |  |  |
| a41 | 5 | F | 2x | Southern slope | - |  |  |  |
| a42 | 5 | F | 2x | Southern slope | - |  |  |  |
| a43 | 5 | F | 2x | Southern slope | - |  |  |  |
| a44 | 6 | F | 2x | Southern slope | - |  |  |  |
| a45 | 11 | F | 2x | Southern slope | - |  |  |  |
| a46 | 8 | F | 2x | Southern slope | - |  |  |  |
| a47 | 9 | F | 2x | Southern slope | - |  |  |  |
| a48 | 7 | F | 2x | Southern slope | - |  |  |  |
| a49 | 5 | F | 2x | Southern slope | - |  |  |  |
| a50 | 8 | F | 2x | Southern slope | - |  |  |  |
| a51 | 5 | F | 2x | Southern slope | - |  |  |  |
| a52 | 9 | F | 2x | Southern slope | - |  |  |  |
| a53 | 10 | F | 2x | Southern slope | - |  |  |  |
| a54 | 9 | F | 2x | Southern slope | - |  |  |  |
| a55 | 7 | F | 2x | Southern slope | - |  |  |  |
| a56 | 8 | F | 2x | Southern slope | - |  |  |  |
| a57 | 6 | N | 2x | Southern slope | 201705017 |  |  |  |
| a58 | 8 | N | 2x | Southern slope | 201705018 |  |  |  |
| a59 | 14 | N | 2x | Southern slope | 201705019 |  |  |  |
| a60 | 5 | N | 2x | Southern slope | 201705020 |  |  |  |
| a61 | 7 | N | 2x | Southern slope | 201705021 |  |  |  |
| a62 | 3 | N | 2x | Southern slope | 201705022 |  |  |  |
| a63 | 5 | N | 2x | Southern slope | 201705023 |  |  |  |
| a64 | 8 | N | 2x | Southern slope | 201705024 |  |  |  |
| a65 | 7 | N | 2x | Southern slope | - |  |  |  |
| a66 | 6 | N | 2x | Southern slope | - |  |  |  |
| a67 | 8 | N | 2x | Southern slope | - |  |  |  |
| a68 | 14 | N | 2x | Southern slope | - |  |  |  |
| a69 | 5 | N | 2x | Southern slope | - |  |  |  |
| a70 | 7 | N | 2x | Southern slope | - |  |  |  |
| a71 | 3 | N | 2x | Southern slope | - |  |  |  |
| a72 | 5 | N | 2x | Southern slope | - |  |  |  |
| a73 | 8 | N | 2x | Southern slope | - |  |  |  |
| a74 | 7 | N | 2x | Southern slope | - |  |  |  |
| a75 | 6 | N | 2x | Southern slope | - |  |  |  |
| a76 | 8 | N | 2x | Southern slope | - |  |  |  |
| a77 | 14 | N | 2x | Southern slope | - |  |  |  |
| a78 | 5 | N | 2x | Southern slope | - |  |  |  |
| a79 | 7 | N | 2x | Southern slope | - |  |  |  |
| a80 | 3 | N | 2x | Southern slope | - |  |  |  |
| a81 | 5 | N | 2x | Southern slope | - |  |  |  |
| a82 | 8 | N | 2x | Southern slope | - |  |  |  |
| a83 | 9 | N | 2x | Southern slope | - |  |  |  |
| a84 | 7 | N | 2x | Southern slope | - |  |  |  |
| a85 | 7 | N | 2x | Southern slope | - |  |  |  |
| a86 | 6 | N | 2x | Southern slope | - |  |  |  |
| a87 | 10 | N | 2x | Southern slope | - |  |  |  |
| a88 | 6 | N | 2x | Southern slope | - |  |  |  |
| a89 | 8 | N | 2x | Southern slope | - |  |  |  |
| a90 | 7 | N | 2x | Southern slope | - |  |  |  |
| a91 | 7 | N | 2x | Southern slope | - |  |  |  |
| a92 | 10 | N | 2x | Southern slope | - |  |  |  |
| a93 | 5 | N | 2x | Southern slope | - |  |  |  |
|  |  |  |  | Northern slope |  | 109°24′ | 32°03′ | 1780 |
| Bc1 | 7 | F | 4x | Northern slope | 201705025 |  |  |  |
| Bc2 | 8 | F | 4x | Northern slope | 201705026 |  |  |  |
| Bc3 | 9 | F | 4x | Northern slope | 201705027 |  |  |  |
| Bc4 | 8 | F | 4x | Northern slope | 201705028 |  |  |  |
| Bc5 | 8 | F | 4x | Northern slope | 201705029 |  |  |  |
| Bc6 | 6 | F | 4x | Northern slope | 201705030 |  |  |  |
| Bc7 | 5 | F | 4x | Northern slope | 201705031 |  |  |  |
| Bc8 | 7 | F | 4x | Northern slope | 201705032 |  |  |  |
| Bc9 | 10 | F | 4x | Northern slope | 201705033 |  |  |  |
| Bc10 | 8 | F | 4x | Northern slope | 201705034 |  |  |  |
| Bc11 | 8 | F | 4x | Northern slope | 201705035 |  |  |  |
| Bc12 | 7 | F | 4x | Northern slope | 201705036 |  |  |  |
| Bc13 | 7 | F | 4x | Northern slope | 201705037 |  |  |  |
| Bc14 | 6 | F | 4x | Northern slope | 201705038 |  |  |  |
| Bc15 | 8 | F | 4x | Northern slope | 201705039 |  |  |  |
| Bc16 | 9 | F | 4x | Northern slope | 201705040 |  |  |  |
| Bc17 | 8 | F | 4x | Northern slope | - |  |  |  |
| Bc18 | 6 | F | 4x | Northern slope | - |  |  |  |
| Bc19 | 8 | F | 4x | Northern slope | - |  |  |  |
| Bc20 | 5 | F | 4x | Northern slope | - |  |  |  |
| Bc21 | 6 | F | 4x | Northern slope | - |  |  |  |
| Bc22 | 8 | F | 4x | Northern slope | - |  |  |  |
| Bc23 | 5 | F | 4x | Northern slope | - |  |  |  |
| Bc24 | 6 | F | 4x | Northern slope | - |  |  |  |
| Bc25 | 7 | F | 4x | Northern slope | - |  |  |  |
| Bc26 | 10 | F | 4x | Northern slope | - |  |  |  |
| Bc27 | 6 | F | 4x | Northern slope | - |  |  |  |
| Bc28 | 9 | F | 4x | Northern slope | - |  |  |  |
| Bc29 | 10 | F | 4x | Northern slope | - |  |  |  |
| Bc30 | 9 | F | 4x | Northern slope | - |  |  |  |
| Bc31 | 8 | F | 4x | Northern slope | - |  |  |  |
| Bc32 | 7 | F | 4x | Northern slope | - |  |  |  |
| Bc33 | 6 | F | 4x | Northern slope | - |  |  |  |
| Bc34 | 8 | F | 4x | Northern slope | - |  |  |  |
| Bc35 | 9 | F | 4x | Northern slope | - |  |  |  |
| Bc36 | 6 | F | 4x | Northern slope | - |  |  |  |
| Bc37 | 9 | F | 4x | Northern slope | - |  |  |  |
| Bc38 | 8 | F | 4x | Northern slope | - |  |  |  |
| Bc39 | 6 | F | 4x | Northern slope | - |  |  |  |
| Bc40 | 8 | F | 4x | Northern slope | - |  |  |  |
| Bc41 | 8 | F | 4x | Northern slope | - |  |  |  |
| Bc42 | 6 | F | 4x | Northern slope | - |  |  |  |
| Bc43 | 8 | F | 4x | Northern slope | - |  |  |  |
| Bc44 | 5 | F | 4x | Northern slope | - |  |  |  |
| Bc45 | 6 | F | 4x | Northern slope | - |  |  |  |
| Bc46 | 8 | F | 4x | Northern slope | - |  |  |  |
| Bc47 | 5 | F | 4x | Northern slope | - |  |  |  |
| Bc48 | 6 | F | 4x | Northern slope | - |  |  |  |
| Bc49 | 7 | F | 4x | Northern slope | - |  |  |  |
| Bc50 | 10 | F | 4x | Northern slope | - |  |  |  |
| Bc51 | 6 | F | 4x | Northern slope | - |  |  |  |
| Bc52 | 9 | F | 4x | Northern slope | - |  |  |  |
| Bc53 | 10 | F | 4x | Northern slope | - |  |  |  |
| Bc54 | 9 | F | 4x | Northern slope | - |  |  |  |
| Bc55 | 8 | F | 4x | Northern slope | - |  |  |  |
| Bc56 | 7 | F | 4x | Northern slope | - |  |  |  |
| Bc57 | 6 | F | 4x | Northern slope | - |  |  |  |
| Bc58 | 8 | F | 4x | Northern slope | - |  |  |  |
| Bc59 | 9 | F | 4x | Northern slope | - |  |  |  |
| Bc60 | 6 | F | 4x | Northern slope | - |  |  |  |
| Bc61 | 9 | F | 4x | Northern slope | - |  |  |  |
| Bc62 | 7 | N | 4x | Northern slope | 201705041 |  |  |  |
| Bc63 | 10 | N | 4x | Northern slope | 201705042 |  |  |  |
| Bc64 | 6 | N | 4x | Northern slope | 201705043 |  |  |  |
| Bc65 | 7 | N | 4x | Northern slope | 201705044 |  |  |  |
| Bc66 | 8 | N | 4x | Northern slope | 201705045 |  |  |  |
| Bc67 | 6 | N | 4x | Northern slope | 201705046 |  |  |  |
| Bc68 | 6 | N | 4x | Northern slope | 201705047 |  |  |  |
| Bc69 | 8 | N | 4x | Northern slope | 201705048 |  |  |  |
| Bc70 | 5 | N | 4x | Northern slope | 201705049 |  |  |  |
| Bc71 | 9 | N | 4x | Northern slope | 201705050 |  |  |  |
| Bc72 | 7 | N | 4x | Northern slope | - |  |  |  |
| Bc73 | 7 | N | 4x | Northern slope | - |  |  |  |
| Bc74 | 8 | N | 4x | Northern slope | - |  |  |  |
| Bc75 | 8 | N | 4x | Northern slope | - |  |  |  |
| Bc76 | 7 | N | 4x | Northern slope | - |  |  |  |
| Bc77 | 7 | N | 4x | Northern slope | - |  |  |  |
| Bc78 | 4 | N | 4x | Northern slope | - |  |  |  |
| Bc79 | 7 | N | 4x | Northern slope | - |  |  |  |
| Bc80 | 7 | N | 4x | Northern slope | - |  |  |  |
| Bc81 | 5 | N | 4x | Northern slope | - |  |  |  |
| Bc82 | 10 | N | 4x | Northern slope | - |  |  |  |
| Bc83 | 11 | N | 4x | Northern slope | - |  |  |  |
| Bc84 | 7 | N | 4x | Northern slope | - |  |  |  |
| Bc85 | 10 | N | 4x | Northern slope | - |  |  |  |
| Bc86 | 6 | N | 4x | Northern slope | - |  |  |  |
| Bc87 | 7 | N | 4x | Northern slope | - |  |  |  |
| Bc88 | 8 | N | 4x | Northern slope | - |  |  |  |
| Bc89 | 6 | N | 4x | Northern slope | - |  |  |  |
| Bc90 | 6 | N | 4x | Northern slope | - |  |  |  |
| Bc91 | 8 | N | 4x | Northern slope | - |  |  |  |
| Bc92 | 5 | N | 4x | Northern slope | - |  |  |  |
| Bc93 | 9 | N | 4x | Northern slope | - |  |  |  |
| Bc94 | 7 | N | 4x | Northern slope | - |  |  |  |
| Bc95 | 7 | N | 4x | Northern slope | - |  |  |  |
| Bc96 | 8 | N | 4x | Northern slope | - |  |  |  |
| Bc97 | 8 | N | 4x | Northern slope | - |  |  |  |
| Bc98 | - | - | 4x | Northern slope | - |  |  |  |
| Bc99 | - | - | 4x | Northern slope | - |  |  |  |
| Bc100 | - | - | 4x | Northern slope | - |  |  |  |
| Bc101 | - | - | 4x | Northern slope | - |  |  |  |
| Bc102 | - | - | 4x | Northern slope | - |  |  |  |
| Bc103 | - | - | 4x | Northern slope | - |  |  |  |
| Bc104 | - | - | 4x | Northern slope | - |  |  |  |
| Bc105 | - | - | 4x | Northern slope | - |  |  |  |
| Bc106 | - | - | 4x | Northern slope | - |  |  |  |
| Bc107 | 1 | - | 4x | Northern slope | - |  |  |  |

| **Table S2** Characteristics of microsatellites for Clintonia udensis | | | | |
| --- | --- | --- | --- | --- |
| Locus | Primer sequence (5′-3′) | Anneal Temp Ta (℃) | Number of alleles | |
|  |  |  | 2n | 4n |
| ES07 | F: GGCATCGATGATGAGGACTT | 50.2℃ | 12 | 26 |
|  | R: ACAAGTAGGGCAACGGGTT |  |  |  |
| ES10 | F: TGCTTGAGATGTGCGACGAG | 51.2℃ | 17 | 20 |
|  | R: CAAGAGAATGCGACAAAGC |  |  |  |
| ES11 | F: TGGCTCGAACCTTCTGAGTT | 51.1℃ | 15 | 22 |
|  | R: CCTCGGATTGTTGATCCTGT |  |  |  |
| ES13 | F: AGAGGTGGAACACGGACATC | 51.6℃ | 10 | 8 |
|  | R: GCCCGGATCACTTTCTTCTC |  |  |  |
| ES16 | F: GCTGGAGCAAGAGAAATTGG | 56.0℃ | 19 | 14 |
|  | R: ACATTTGTTGGTCCCTCCTG |  |  |  |
| ES18 | F: AACCTACCTCCACCCCTACC | 51.1℃ | 16 | 21 |
|  | R: AGAATCAGAGAGGCGGAAC |  |  |  |
| ES22 | F: CACCCAGGCGTAAGAAGAAG | 46.6℃ | 16 | 25 |
|  | R: GCACCTGTGTCAACACCCTA |  |  |  |
| ES23 | F: GCAGGAATTCGGCACGAG | 48.0℃ | 6 | 14 |
|  | R: GCCACATTGGACCATAAACC |  |  |  |

| **TableS3** One-way ANOVA for Clintonia udensis | | | | | | | | | | |
| --- | --- | --- | --- | --- | --- | --- | --- | --- | --- | --- |
|  | | | | | | SS | df | MS | F | Significant level |
| Buds distribution on rhizome | Among groups | | Combination | | | 0.4 | 5 | 0.08 | 0.098 | 0.99 |
|  |  |  | Linear term | Weighted | | 0.009 | 1 | 0.009 | 0.011 | 0.918 |
|  |  |  |  | Deviation | | 0.391 | 4 | 0.098 | 0.12 | 0.972 |
|  | Within groups | |  | | | 7.333 | 9 | 0.815 |  |  |
|  | Total | | | | | 7.733 | 14 |  |  |  |
| The ratio of | Among groups | Combination | | | | 0.061 | 5 | 0.012 | 9.546 | 0.005 |
|  |  | Linear term | | | Weighted | 0.013 | 1 | 0.013 | 10.426 | 0.014 |
| dormancy buds |  |  |  |  | Deviation | 0.048 | 4 | 0.012 | 9.326 | 0.006 |
|  | Within groups | | | | | 0.009 | 7 | 0.001 |  |  |
|  | Total | | | | | 0.07 | 12 |  |  |  |
| Rates of active buds and dormancy buds | Among groups | Combination | | | | 0.764 | 5 | 0.153 | 6.147 | 0.017 |
|  |  | Linear term | | | Weighted | 0.131 | 1 | 0.131 | 5.267 | 0.055 |
|  |  |  |  |  | Deviation | 0.633 | 4 | 0.158 | 6.366 | 0.017 |
|  | Within groups | | | | | 0.174 | 7 | 0.025 |  |  |
|  | Total | | | | | 0.938 | 12 |  |  |  |
| Ratio of active buds | Among groups | Combination | | | | 0.072 | 5 | 0.014 | 11.063 | 0.003 |
|  |  | Linear term | | | Weighted | 0.015 | 1 | 0.015 | 11.487 | 0.012 |
|  |  |  |  |  | Deviation | 0.057 | 4 | 0.014 | 10.956 | 0.004 |
|  | Within groups | | | | | 0.009 | 7 | 0.001 |  |  |
|  | Total | | | | | 0.081 | 12 |  |  |  |
| The average number of rhizome buds | Among groups | Combination | | | | 1.69 | 5 | 0.338 | 6.423 | 0.015 |
|  |  | Linear term | | | Weighted | 0.747 | 1 | 0.747 | 14.201 | 0.007 |
|  |  |  |  |  | Deviation | 0.943 | 4 | 0.236 | 4.478 | 0.041 |
|  | Within groups | | | | | 0.368 | 7 | 0.053 |  |  |
|  | Total | | | | | 2.058 | 12 |  |  |  |
